# Control of HIV-1 by multiple immunodominant HIV-1-specific CD8+ T cells in HIV-1-infected Japanese individuals

**DOI:** 10.1186/1742-4690-9-S2-P256

**Published:** 2012-09-13

**Authors:** H Murakoshi, M Koyanagi, H Gatanaga, T Naruto, S Oka, M Takiguchi

**Affiliations:** 1 Center for AIDS Research, Kumamoto University, Kumamoto, Japan; 2 AIDS Clinical Center, National Center for Global Health and Medicine, Tokyo, Japan

## Background

Previous studies of the comprehensive analysis of HIV-1-specific CTL responses in Caucasian and African cohorts demonstrated the association of the CTL responses to HIV-1 Gag protein with the control of HIV-1 replication. However, such analysis in Asian cohorts has not been reported. In the present study, we performed the comprehensive analysis of CD8^+^ T cell responses against 11-mer overlapping HIV-1 Nef, Gag, and Pol peptides in 401 chronically HIV-1 clade B-infected treatment-naive Japanese individuals.

## Methods

The CD8^+^ T cell responses to cocktails of the peptides were evaluated by measuring IFN-g-producing CD8^+^T cells by using ELISPOT assay.

## Results

To clarify CTLs which control HIV-1 infection in this cohort, we statistically analyzed differences of viral load and CD4 counts between responders to each peptide cocktail in each HLA^+^ individuals and non-responders using two-tailed Mann-Whitney’s test. We found that several HLA alleles were significantly correlated with low viral load and high CD4 counts in the responses to 5 Nef, 10 Gag, or 16 Pol cocktails. In these cocktails, we identified 2 Nef, 12 Gag and 7 Pol CTL epitopes restricted by 9 HLA alleles. The breadth of CTL responses to these epitopes was significantly associated with low viral load (p=1.7x10^-10^) and high CD4 counts (p=4.1x10^-13^). The total magnitude of responses to the epitopes was also significantly correlated with low viral load (r=-0.30, p=1.8x10^-9^) and high CD4 counts (r=0.37, p=5.0x10^-14^).

## Conclusion

These results suggest that the CTL responses to these epitopes play an important role in the control of HIV-1 infection in chronically HIV-1-infected Japanese individuals.

